# Understanding brain cancer and exploiting its vulnerabilities

**DOI:** 10.1002/1878-0261.13769

**Published:** 2024-11-25

**Authors:** Paolo Salomoni

**Affiliations:** ^1^ Nuclear Function Group, German Center for Neurodegenerative Diseases (DZNE) Bonn Germany

## Abstract

Brain cancer is one of the most devastating neoplasms affecting both children and adults. Its dismal prognosis has for long‐time discouraged research in this area. However, in the last 10–15 years remarkable progress has been made in our understanding of brain cancer biology, thus showing promise for the identification of new ways to treat these tumors towards the improvement of patients' survival and quality of life. This Thematic Issue on Brain cancers offers a much needed and timely critical overview of the fundamental discoveries in this area of research and which of those are more promising for effective translation into the clinic. Critically, many Reviews from this Issue also provide discussion points on why the field has not progressed as much as it had hoped to. It is important to emphasize however that we are living in exciting times with regards to our ability to translate fundamental findings into the clinic thanks to the enormous technological advances facilitating the study of cancer genomes and the development of new drugs or repurposing existing agents.

## Graphical abstract

Brain cancers display inter‐ and intra‐tumor heterogeneity at the genetic, epigenetic and metabolic level. This extends to the tumor microenvironment (TME), which is believed to fuel cancer progression, but at the same time offers opportunities for therapeutic exploitation. The Thematic Issue on Brain Cancer covers some of the most exciting developments in the area.

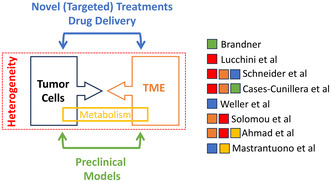

**Abbreviations**
2‐HG: 2‐hydroxyglutarate
ANG2: Angiopoietin 2
BCR‐ABL: breakpoint cluster region‐Abelson
c‐MET: mesenchymal epithelial transition
CML: chronic myelogenous leukemia
CXCL12: CXC ligand 12
CXCR4: C‐X‐C Motif Chemokine Receptor 4
EGFR: epidermal growth factor receptor
GBM: glioblastoma multiforme
IDH: isocitrate dehydrogenase enzyme
LDLR: low‐density lipoprotein receptor
LGNTs: low‐grade neuroepithelial tumors
LRP‐1: LDLR‐related protein‐1
MAPK: mitogen‐activated protein kinase
MHC‐II: major histocompatibility complex‐II
MMR: mismatch repair
NGS: Next generation sequencing
PMMRDIA: primary mismatch repair deficient‐IDH‐mutated astrocytomas
PTMs: posttranslational modifications
TAM: tumor‐associated macrophage
TME: tumor microenvironment
TMs: tumor microtubes

Brain cancers are a group of heterogeneous tumor entities affecting children to adults, representing one of the main causes of death in the working‐age population. This is due to their intrinsic aggressiveness and heterogeneity, underlying their refractoriness to therapy and rapid progression even after tumor debulking and aggressive chemo/radiotherapy. Research in this area has been hampered for many years by limited funding and uncoordinated research efforts. However, thanks to enhanced advocacy/awareness and progressively increasing funding, we are gaining key insights into how these tumors develop, which are the main drivers and what role the tumor microenvironment (TME) plays. Our ability to identify genomic changes driving these neoplasms show promise for developing targeted approaches as part of personalized medicine efforts. Another area of poor progress was preclinical modeling, which in turn hampered progress in our understanding of tumor biology. However, substantial advances have been made in this area with regards to the generation of genetically modified animal models recapitulating the main histological and molecular features of these tumors.

This collection of Reviews aims at covering the main advances and challenges in this area, from preclinical modeling to identification of novel and targetable tumor vulnerabilities.

## 1 Advances in preclinical modeling

The Review by Brandner et al. [1] provided an extensive overview of the main mouse models for brain cancer, from the dawn of brain cancer preclinical modeling to the recent advances in gene editing. This Review would serve both the neophyte to the field and the expert glioma scientist or clinician looking for key details on the main models available. The description of early models is particularly fascinating, as it provides key details on early attempts while on the other hand highlights how research has evolved in this area supported by the enormous progress in the application of genetic engineering in animals. Nonetheless, a degree of caution comes from the understanding of how little progress has been made with respect to developing new therapies. Animal models have taught us a lot about brain cancer biology, but many of them fail to fully mimic the inter‐ and intratumor heterogeneity of these neoplasms as well as the complex interaction between tumor cells and the TME.

## 2 Molecular and functional heterogeneity

Heterogeneity of brain cancer is caused by both genetic and epigenetic diversity. The latter represents the main theme in the Review by Marino et al. [2], which provides a comprehensive overview of epigenetic alterations in glioblastoma multiforme (GBM) and how they can be used to classify these neoplasms. The authors for instance highlight how DNA methylation can be used to infer the composition of the TME, and more specifically predict the infiltration by lymphocytes. The roles of histone posttranslational modifications (PTMs) are also discussed, citing several exciting studies on GBM‐initiating cells and their multi‐histone PTM profiles. In this respect, the activation status of enhancers and other regulatory elements have been linked to different GBM states. Furthermore, non‐coding RNAs such as microRNAs and long non‐coding RNAs are emerging as potential markers of progression or response to therapy. Together, these studies point at the importance of these epigenetic mechanisms in driving molecular heterogeneity. However, how these molecular perturbations are linked to different behaviors of tumor cells remains incompletely understood. This is the focus of the Review article by Schneider et al. [3] Seminal work by several laboratories in the field has led to the identification of extensive tumor networks, predominantly subset of GBM tumors that do not carry mutations in the isocitrate dehydrogenase enzyme (IDH, covered below). These networks can be classified as *homotypical* and *heterotypical*, depending on whether tumor cells only interact via each other or with other cells within the TME. With respect to homotypical interactions, non‐connecting tumor microtubes (TMs) are found more at the tumor margins and are associated with sensitivity to therapy and invasive capacity. In contrast the connecting TMs are found predominantly at the tumor core and are linked to resistance to therapy. Then, the Review covers work on the potential mechanisms regulating formation and maintenance of these networks, such as the cytoskeletal component, connexin 43. However, the most exciting development in this field is the discovery of network/synaptic interactions between neurons and tumor cells, and how such network can reciprocally influence the function of the connected cells. It is fascinating how these interactions can involve bidirectional regulation between tumor cells and astrocytes, in addition to neurons. The last part reports on what advances are being made on our ability to cause network disconnection in therapeutic settings. These approaches range from the surgical domain to pharmacological intervention aimed at producing morphological and/or functional disruption of tumor networks. One of the FDA approved drugs that are emerging is meclofenamate, an agent investigated by the authors of this Review and others in the field. The combination of meclofenamate (to disrupt tumor cell‐to‐tumor cell interactions) and Perampanel (to interfere with tumor cell‐to‐neuron synapses) is being currently tested in trials.

Tumor networks in the context of other brain cancer entities is the subject of the Review written by Gielen et al. [4] The main topic is low‐grade neuroepithelial tumors (LGNTs) with glioneuronal histology, which are associated with pharmacological treatment‐resistant epilepsy and for which no specific treatment is yet available. This Review offers a much‐needed overview of the classification of these tumor entities based on histological and molecular criteria. One of the reasons for our previously limited understanding of these tumors was the lack of suitable preclinical models. The authors then cover the development of *in‐utero* electroporation‐based models, which have been pioneered by them and others. These models recapitulate the key features of these neoplasms, in particular the convulsive activity of their glioma/neuronal networks and show promise for an improved grasp of the key biological features of LGNTs. In particular, work from the authors of this Review and others has provided key insights into how tumor cells influence neuro/glial networks. However, less is known on whether these interactions are bidirectional, as it has been described for other gliomas covered in the Review by Schneider et al., for instance.

## 3 Precision oncology in GBM

The Reviews by Schneider and Gielen set the scene for a comprehensive appraisal of precision oncology in glioblastoma by Herrlinger et al. [5] The area of targeted therapy started with much excitement and expectation around oncogene‐targeting treatment for tumors driven by a single tumor driver, such as breakpoint cluster region‐Abelson (BCR‐ABL) in chronic myelogenous leukemia (CML) and BRAF in melanoma. However, the appreciation of intra‐tumor heterogeneity along with the failure of many targeted agents to treat many cancers has somewhat dampened this excitement. This Review covers the current status and directions in the area of glioblastoma precision oncology. It Reviews preclinical research and clinical applications of agents targeting well‐known tumor drivers such as mutant IDH, epidermal growth factor receptor (EGFR) and emerging ones, such mesenchymal epithelial transition (c‐MET). Downstream effectors and the corresponding genetic alterations found in glioblastomas are covered, including components of the PI3K pathway. Despite the shortcomings of several drugs in the clinical setting, some of the targeted agents with previous target verification show promise. A compelling example is the recent clinical trial results from the use of mutant IDH‐specific inhibitor Vorasidenib in grade‐2 gliomas. Furthermore, therapies targeting BRAF and MEK in patients displaying constitutive mitogen‐activated protein kinase (MAPK) pathway activation showed promise and may be considered part of the standard of care for these neoplasms. The Review then covers mostly unsuccessful trials using inhibitors against other main glioblastoma drivers, such as EGFR. These approaches suffer from a lack of appreciation of how intra‐tumor heterogeneity with respect to receptor tyrosine kinase gains plays a key role for the emergence of targeted‐therapy resistant clones. Approaches such as the one targeting both the tumor driver (e.g., BRAF mutations) and downstream tumor‐driving signaling (e.g., MEK) show much better promise for efficacy in trials. Authors describe how NGS‐based molecular‐guided GBM therapy could form part of personalized medicine‐driven clinical studies. This is an area suffering from a paucity of studies, and within those few, only a small fraction of next generation sequencing (NGS)‐screened patients receives treatment. The Review ends with a stimulating section on how precision oncology is moving beyond tumor cell targets into the realm of tumor microenvironment (TME) targeting.

In their Review, Herrlinger et al. cover three main areas: anti‐angiogenic therapy, immunotherapy and therapy targeting tumor networks (complementary to the Review by Schneider et al. covered above). It is important to note that some of the same researchers authoring this and the previous Review have been involved in exciting work aimed at exploiting TME vulnerabilities in glioblastoma, thus representing a first‐hand experience in this exciting area of clinical investigation. Finally, I would like to highlight the number of trials using inhibitors of C‐X‐C Motif Chemokine Receptor 4 (CXCR4) or its ligand CXC ligand 12 (CXCL12), which promote macrophage exclusion from the tumor, an exciting approach for remodeling the TME for reducing tumor burden.

## 4 The immune‐tumor microenvironment of glioblastoma

Tumor‐associated macrophages (TAMs) represent the main topic of the Review authored by Bulstrode et al. [6] TAMs within glioblastomas often carry immune‐suppressive anti‐inflammatory features that are believed to sustain tumor growth and limit immune reactions against tumor cells. However, immune‐therapies devised to target these tumor‐sustaining features and to unleash the immune system, have to‐date failed in gliomas, unlike in other neoplasms where they have achieved striking success. Therefore, it is fundamental to learn more about TAMs and their features within gliomas, in order to devise new ways to engage the immune system against these devastating tumors. The Review focuses on the use of single‐cell and spatial multi‐omic approaches, which bear promise to improve modeling and ultimately lead to improved targeting of tumor/immune cell interactions. A comprehensive overview of the current knowledge in the area of microglia diversity in development, disease and health provides intriguing insights for instance into gender differences applying to those TAMs that are defined as myeloid‐derived suppressor cells. This section is followed by a critical assessment of differences between TAM states *in vitro* and *in vivo*, in particular on the long‐lasting and long‐debated M0 to M2 states. The advent of single‐cell and spatial molecular annotation in patient and models has revolutionized the field, particularly around TME heterogeneity, which was comprehensively covered by this Review. This section provides the reader with key insights into how the field has progressed and what are the most interesting directions and developments. In particular authors discuss how molecular features of TAMs change depending on the location within the core or margins of the tumor, and on the differences and similarities of TAMs across different tumors. Additionally, the Review discusses how glioma cells and TAMs interact and the understanding of this topic, especially in the context of how the genetic makeup of tumors influences TAMs. The two final sections are on the need to develop models based on human TAM/glioma interaction and, importantly on the current state‐of‐the art and future for targeting TAMs for treatment. There is much promise for how innate immune cells could be targeted for improving survival and therapy response in glioma/glioblastoma.

Tumor immunology along with DNA repair deficiency are the topics of the Review article by Pfister et al. [7] In particular, the authors focus on primary mismatch repair deficient‐IDH‐mutated astrocytomas (PMMRDIA). These tumors were originally described as a distinct group of gliomas, separate from other IDH‐mutant gliomas, including those with secondary mismatch repair (MMR) deficiency. PMMRDIA patients do not respond to standard of care temozolomide treatment as well as to immune checkpoint blocker therapy, thus representing a therapeutic challenge. The Review provides an extensive overview of the mechanisms of action of 2‐hydroxyglutarate (2‐HG) and then critically assesses the current knowledge on how IDH mutations and 2‐HG are linked to immune suppression. This assessment was set as a systematic Review, with a protocol registered in the International Prospective Register of Systematic Reviews (PROSPERO). It provides details on the search and review strategies, with respect to eligible studies and inclusion/exclusion criteria. The results of this literature search are very interesting, as they point at multiple mechanisms underlying immune‐suppression in IDH‐mutated gliomas, such as lower tumor mutation burden, reduced major histocompatibility complex‐II (MHC‐II) expression, suppression of immune cell chemotaxis and T‐cell immunity, escape from natural killer cell immunity and others.

## 5 Exploiting glioblastoma metabolism for therapy

Finally, I would like to highlight an exciting piece by Battaglia et al. [8], which epitomizes the importance of efforts coming from researchers that operate within areas that are not directly linked to brain cancer research. This field indeed requires out‐of‐the‐box thinking given that progress in the last decades has been somewhat limited and this type of cancer remains incurable. This Review article combines what is known about a particular family of receptors regulating the blood–brain barrier, their roles in brain cancer and their potential as therapeutic targets. The low‐density lipoprotein receptor (LDLR) family includes LDLR and LDLR‐related protein‐1 (LRP‐1), which play a critical role in the cellular uptake of various molecules. They are mostly studied for their ability to bind to and mediate the internalization of LDL cholesterol, a function enhanced in several cancers, including glioblastoma, because of their addiction to cholesterol. Due to the presence of the BBB, it has been postulated that LDLRs represent one of the main mechanisms for providing cholesterol to glioblastoma cells for their proliferation. Interestingly, these receptors activate a number of key signaling pathways, previously linked to glioblastoma, such as the Wnt/beta‐catenin pathway. However, LDLRs are expressed not only in cancer cells but also by endothelial cells, and recent studies have described their targeting via nanoparticles modified to encapsulate drugs like sorafenib. Of note is the emergence of LRP‐1 as target to cross the blood brain barrier and deliver drugs to GBM cells. For instance, conjugates with the LRP‐1 target, Angiopoietin 2 (ANG2) are already in clinical trials for brain cancers. Overall, this highlights the potential of nanotechnology to overcome the limitations of current therapies against glioblastoma.

## 6 Conclusions

This Thematic Issue on Brain Cancers offers a comprehensive overview of how brain cancer research has progressed in the last couple of decades and what are the main discoveries with respect to fundamental science and clinical translation. At the same time, it conveys a strong message that substantial efforts are needed to bring new and provocative ideas to the field, in particular for what concerns an improved understanding of tumor biology and vulnerabilities that could be targeted clinically. One major area for investment is on how TME/tumor cell dynamics influences tumor progression and could be exploited for improving response to therapy and ultimately, patients' survival.

## Conflict of interest

The authors declare no conflict of interest.
